# Single-cell computational analysis of light harvesting in a flat-panel photo-bioreactor

**DOI:** 10.1186/s13068-018-1147-3

**Published:** 2018-05-26

**Authors:** Varun Loomba, Gregor Huber, Eric von Lieres

**Affiliations:** 1 Forschungszentrum Jülich GmbH, Institute of Bio- and Geosciences, IBG-1: Biotechnology, Wilhelm-Johnen-Straße, 52428 Jülich, Germany; 2 Forschungszentrum Jülich GmbH, Institute of Bio- and Geosciences, IBG-2: Plant Sciences, Wilhelm-Johnen-Straße, 52428 Jülich, Germany

**Keywords:** Microalgae, Hydrodynamics, Computational fluid dynamics, Radiative transfer equation, Particle tracing

## Abstract

**Background:**

Flat-panel photo-bioreactors (PBRs) are customarily applied for investigating growth of microalgae. Optimal design and operation of such reactors is still a challenge due to complex non-linear combinations of various impact factors, particularly hydrodynamics, light irradiation, and cell metabolism. A detailed analysis of single-cell light reception can lead to novel insights into the complex interactions of light exposure and algae movement in the reactor.

**Results:**

The combined impacts of hydrodynamics and light irradiation on algae cultivation in a flat-panel PBR were studied by tracing the light exposure of individual cells over time. Hydrodynamics and turbulent mixing in this air-sparged bioreactor were simulated using the Eulerian approach for the liquid phase and a slip model for the gas phase velocity profiles. The liquid velocity was then used for tracing single cells and their light exposure, using light intensity profiles obtained from solving the radiative transfer equation at different wavelengths. The residence times of algae cells in defined dark and light zones of the PBR were statistically analyzed for different algal concentrations and sparging rates. The results indicate poor mixing caused by the reactor design which can be only partially improved by increased sparging rates.

**Conclusions:**

The results provide important information for optimizing algal biomass productivity by improving bioreactor design and operation and can further be utilized for an in-depth analysis of algal growth by using advanced models of cell metabolism. 
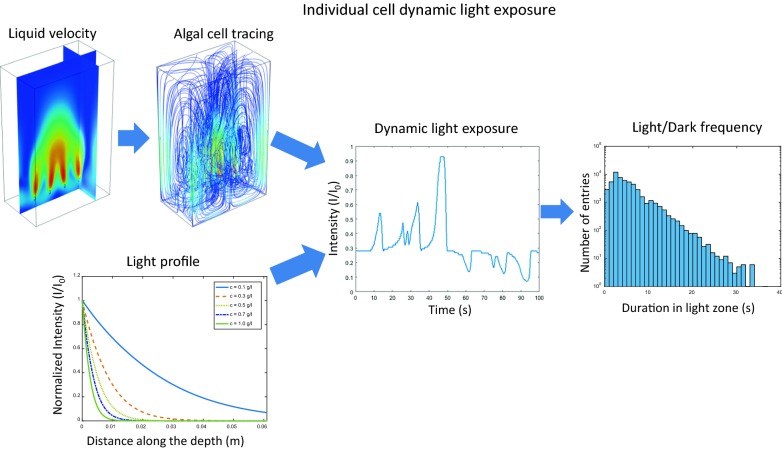

## Background

Microalgae are known for their ability to produce a large number of valuable products by converting CO_2_ via photosynthesis, making their production significant in various fields including the chemical industry, food and agriculture industry, cosmetics etc. [1, 2]. They not only produce valuable products but also reduce excess CO_2_ from the environment. Higher plants are also capable of manufacturing many of these hydrocarbon molecules, but at optimal conditions microalgae are potentially able to produce more product per unit area, making them valuable and interesting to study [3].

Microalgae are usually cultivated in closed PBRs or in open raceway ponds [4]. Here, we focus on closed PBRs because they offer better control of environmental conditions and are less prone to contamination [5]. PBRs can be of different shapes and sizes, e.g., tubular, bubble columns, flat-panel, and airlift reactors [6–9]. Most of these are sparged with air bubbles with elevated CO_2_ concentrations, which serves for two purposes: firstly to improve the mixing of algal cells in the reactor, and secondly to increase the mass transfer of CO_2_ from gas to liquid phase by increasing the interfacial area between gas and liquid in the PBR and thus improving the rate of photosynthesis [10, 11].

A major limiting factor in the photosynthetic process is availability of light to the algal cells [12]. In a PBR, light is absorbed and scattered by algae. The amount of light attenuation depends on the path length into the reactor, wavelength of light, algal cell concentration and pigment composition of the cultivated strain. At high concentrations, satisfying photosynthetic rates can only be achieved within a PBR geometry that minimizes the length of the light path inside the reactor. Different illumination schemes, i.e., internal or external illumination and flashing light effects, have been studied to improve the light distribution inside PBRs [13–18]. The incident light intensity needs to be sufficiently high to support growth inside a given reactor geometry, but on the other hand should not be too high to avoid photo-inhibition for algae cells at the reactor surface [12].

At high cell concentrations, the algae near the light source can shield other cells in the interior of the reactor from light and thus reduce photosynthetic efficiency. Hence, good mixing is required so that all algae cells receive similar amounts of light. Yet, the impact of hydrodynamics on PBR efficiency is still under debate [19]. Pruvost et al. [19] have shown that average growth does not depend on hydrodynamics, except for light/dark cycles effects, because all light entering the PBR will be photosynthetically converted independently of algae movement, at least at concentrations high enough to ensure that no light can exit the system.

The present study aims at investigating the relationship of hydrodynamics and light reception by single algal cells in more detail, on a single-cell level beyond averages of light intensity, with a focus on the statistical distribution of light exposure of algal cells as they move inside the PBR. Our working hypothesis was that the dynamic light exposure of individual algal cells depends on the gas flow rate, but only to a certain extent, depending on the design of the PBR. The hydrodynamics in a lab scale sparged flat-panel PBR were simulated using computational fluid dynamics (CFD) in order to analyze velocity and pressure profiles of liquid and gas phases. A flat-panel geometry was chosen because it is well suited for fundamental investigations such as the determination of algal growth characteristics [20–23]. In a next step, the velocity profiles were used for tracing the paths of algal cells in the studied PBR. These algal cell traces were combined with light intensity profiles calculated using the radiative transfer equation (RTE) to obtain the history of light exposure of single algal cells.

## Methods

### Reactor dimensions

The PBR simulated in this study is a flat-panel PBR, similar to those produced by Photons Systems Instruments (PSI), Brno, Czech Republic [20]. The dimensions of the PBR are 10.34 × 6.1 × 19.83 cm with a total capacity of 1.25 L, but it is typically filled only up to 1 L. A panel of light emitting diodes (LEDs) provides light from one side (10.34 × 9.83 cm) of the PBR. The light intensity can be controlled. By PBR design, the light is homogeneously distributed and perpendicularly incident to the reactor surface. A gas inlet pipe passes through the PBR with four gas inlet holes to sparge air into the system at a constant volumetric rate, typically around 0.5 L/min (8.33 × 10^−6^ m^3^/s). This pipe is excluded from the simulations to simplify the model and speed up simulations. However, the positions of the four inlet holes in the simulations are the same as in the inlet pipe. The holes are modeled as circular in shape with a diameter of 0.8 mm.

### Hydrodynamics

For simulating the hydrodynamics of the system, liquid and gas phases were considered. The algal cells are considered to be part of the liquid phase in the PBR as the algal concentration is always low enough to not change the physical properties of the liquid. Air is sparged from the inlet pipe, creating a turbulent motion. Navier–Stokes equations were solved for the liquid phase using the Eulerian approach. For the gas phase velocity profiles, a Lagrangian approach was used (i.e., applying a slip model), which is valid under the assumption that the gas phase volume fraction is much smaller than the liquid phase volume fraction. Based on the same assumption, coagulation and breakage of bubbles can be neglected [24]. Both liquid and gas phases share the same pressure field. The boundary conditions at the walls of the PBR were defined as no-slip, i.e., the liquid directly at the wall surface has zero velocity. In order to reduce simulation time, the air–water interface at the top of the PBR was not considered as a free surface, but a slip boundary condition was applied for the liquid phase and an outlet for the gas phase. The flow equations describe momentum transport, Eq. 1a, and continuity, Eq. 1b [25].

1a $$\varphi_{L} \rho_{L} \frac{{\partial {\mathbf{u}}_{L} }}{\partial t}\, + \, \varphi_{\text{L}} \rho_{\text{L}} {\mathbf{u}}_{\text{L}} \cdot \nabla {\mathbf{u}}_{\text{L}} \, = \, - \,\nabla p\, + \,\nabla \cdot \left[ {\varphi_{\text{L}} \left( {\mu_{\text{L}} \, + \,\mu_{\text{T}} } \right)\left( {\nabla {\mathbf{u}}_{\text{L}} \, + \,\nabla u_{\text{L}}^{\text{T}} - \frac{2}{3}\left( {\nabla \cdot {\mathbf{u}}_{\text{L}} } \right)I} \right)} \right]\, + \,\varphi_{\text{L}} \rho_{\text{L}} g$$

1b $$\frac{\partial }{\partial t}\left( {\rho_{\text{L}} \varphi_{\text{L}} \, + \,\rho_{\text{g}} \varphi_{\text{g}} } \right)\, + \,\nabla \cdot \left( {\rho_{\text{L}} \varphi_{\text{L}} {\mathbf{u}}_{\text{L}} \, + \,\rho_{\text{g}} \varphi_{\text{g}} {\mathbf{u}}_{\text{g}} } \right)\, = \,0$$

In Eq. 1a, b, *φ*_L_, **u**_L_, *ρ*_L_, and *μ*_L_ are the liquid phase volume fraction, time averaged velocity, density, and viscosity, respectively. *φ*_g_, **u**_g_ and *ρ*_g_ are the gas phase volume fraction, time averaged velocity, and density, respectively. *p* is the pressure; *g* (≈ 9.8 m/s^2^) is the acceleration due to gravity, and *μ*_*T*_ is the liquid phase turbulent viscosity. The liquid density (*ρ*_L_ = 1000 kg/m^3^), liquid viscosity ($\mu_{\text{L}} = 1$ mPa s), and gas density (*ρ*_g_ = 0.85 kg/m^3^) remain constant during the simulation.

The velocity of the gas phase was calculated by summing up drift velocity, **u**_drift_, slip velocity, **u**_slip_, and liquid phase velocity, **u**_L_:

2a $${\mathbf{u}}_{\text{g}} \, = \,{\mathbf{u}}_{\text{L}} \, + \, {\mathbf{u}}_{\text{slip}} \, + \, {\mathbf{u}}_{\text{drift}}.$$

**u**_slip_ is the relative velocity between the liquid and the gas phase, which was calculated using the definition of drag force:

2b $$\nabla p\, = \, - \,C_{\text{D,b}} \frac{{3\rho_{\text{L}} }}{{4d_{\text{b}} }}\left| {{\mathbf{u}}_{\text{slip}} } \right|{\mathbf{u}}_{\text{slip}}.$$

Here, *d*_b_ is the gas bubble diameter, which was assumed constant at 3 mm. *C*_D,b_ is the dimensionless drag coefficient given by

2c $$C_{\text{D,b}} \, = \,\frac{24}{{{\text{Re}}_{\text{b}} }}\left( {1\, + \,0.15{\text{Re}}_{\text{b}}^{0.687} } \right).$$

Re_b_ is the bubble Reynolds number. The drift velocity was calculated by

2d $${\mathbf{u}}_{\text{drift}} \, = \, - \,\frac{{\mu_{\text{L}} + \mu_{T} }}{{\rho_{\text{L}} }}\frac{{\nabla \varphi_{g} }}{{\varphi_{g} }}.$$

Turbulent flow is described using the standard *k* − *ɛ* model [26], where *k* is the turbulent kinetic energy and *ɛ* is the turbulent energy dissipation rate. *k* was calculated by

3a $$\rho_{\text{L}} \frac{\partial k}{\partial t}\, + \, \rho_{\text{L}} {\mathbf{U}}_{\text{L}} \cdot \nabla k\, = \, \nabla \cdot \left[ {\left( {\mu_{L} \, + \,\frac{{\mu_{T} }}{{\sigma_{k} }}} \right)\nabla k} \right]\, + \,P_{k} \, - \,\rho_{L} \varepsilon \, - \,S_{k}.$$

**U**_L_ is the average liquid phase velocity; *P*_*k*_ is a production term given by

3b $$P_{k} \, = \, \mu_{T} \left( {\nabla {\mathbf{U}}_{\text{L}} :\left( {\nabla {\mathbf{U}}_{\text{L}} \, + \, \left( {\nabla {\mathbf{U}}_{\text{L}} } \right)^{T} } \right)\, - \, \frac{2}{3}\left( {\nabla \cdot {\mathbf{U}}_{\text{L}} } \right)^{2} } \right)\, - \, \frac{2}{3}\rho_{L} k\nabla \cdot {\mathbf{U}}_{\text{L}}.$$

*S*_*k*_ is a source term which accounts for the bubble-induced turbulence:

3c $$S_{k} \, = \, - \,C_{k} \varphi_{g} \nabla p \cdot u_{\text{slip}}.$$

Turbulent viscosity was modeled by [27]

3d $$\mu_{T} \, = \, \rho_{l} C_{\mu } \frac{{k^{2} }}{\varepsilon }.$$

The turbulent energy dissipation rate was estimated by

3e $$\rho_{\text{L}} \frac{\partial \varepsilon }{\partial t}\, + \, \rho_{\text{L}} {\mathbf{U}}_{\text{L}} \, \cdot \,\nabla \varepsilon \, = \, \nabla \cdot \left[ {\left( {\mu_{\text{L}} \, + \,\frac{{\mu_{T} }}{{\sigma_{\varepsilon } }}} \right)\nabla \varepsilon } \right]\, + \,C_{\varepsilon 1} \frac{\varepsilon }{k}P_{k} \, - \,C_{\varepsilon 2} \rho_{\text{L}} \frac{{\varepsilon^{2} }}{k}\, + \, C_{\varepsilon } S_{k} \frac{\varepsilon }{k}.$$

The constants are *C*_*k*_ = 0.6, *C*_*ɛ*1_ = 1.44, *C*_*ɛ*2_ = 1.92, *C*_*ɛ*_ = 1.4, *σ*_*k*_ = 1, *σ*_*ɛ*_ = 1.3, and *C*_*μ*_ = 0.09. The hydrodynamic simulations were performed with COMSOL 5.2a using the turbulent bubbly flow model in the CFD module.

### Particle tracing

The algal cells move inside the reactor due to the drag force of the liquid phase. Given the initial position of an algal cell, the path of that cell can be tracked over time based on the previously determined velocity profiles of the liquid phase, using the particle tracing module of COMSOL 5.2a. The momentum of the particle was calculated by the second law of Newton and is equal to the sum of all the forces acting on the algae [28]:

4a $$\frac{{{\text{d}}m_{p} {\mathbf{v}}}}{{{\text{d}}t}}\, = \,F_{\text{D}} \, + \,F_{g} \, + \,F_{\text{ext}}.$$

In Eq. 4a, *m*_*p*_ is the mass of the particle; **v** is the particle velocity field; *F*_*D*_ is the drag force on the particle; *F*_*g*_ is the gravitational force, and *F*_ext_ describes any external force acting on the particle. The size of the algal cells is small and the volume fraction of the algae is low enough not to affect the flow profiles of the liquid phase. Therefore, a Lagrangian approach was used for the algal cells such that there is only one-way coupling between the liquid and the algal cells. The density of the algal cells can be considered the same as the liquid phase. The effect of gravitational force can be neglected and there is no external force. Consequently, only the drag force governs the movement of the particles:

4b $$F_{\text{D}} \, = \, \left( {\frac{1}{{\tau_{p} }}} \right)m_{\text{p}} \left( {{\mathbf{u}}\, - \,{\mathbf{v}}} \right).$$

Here, *τ*_p_ is the particle velocity response time, and **u** is the liquid velocity field. The Schiller–Naumann drag law [29] was used to estimate the particle velocity response:

4c $$\tau_{\text{p}} \, = \, \frac{{4\rho_{\text{p}} d_{\text{p}}^{ 2} }}{{3\mu_{\text{L}} C_{\text{D,p}} {\text{Re}}_{\text{p}} }}.$$

In Eq. 4c, *ρ*_p_ and *d*_p_ are the particle density and diameter, respectively; Re_p_ is the particle Reynolds number, and *C*_D,p_ is the particle drag coefficient, calculated in analogy to Eq. 2c. In this study, the diameter of algal cells was taken as 7 µm [30]. A bouncing boundary condition was applied to all the surfaces of the PBR for the algal cells, i.e., the algal cells bounce back at the same angle of reflectance as the angle of incidence.

### Light Intensity simulation

The decrease of photosynthetically active light with increasing concentration of algae and increasing distance from the irradiated surface of the PBR was calculated using the radiative transfer equation (RTE) [31], which takes into account both absorption and scattering of light to calculate light intensities inside the PBR at a given initial irradiation intensity [32]. The RTE can be written as

5a $$\frac{{{\text{d}}I\left( {\lambda ,s} \right)}}{\text{ds}}\, = \, - \,\kappa I\left( {\lambda ,s} \right)\, - \,\sigma_{\text{s}} I\left( {\lambda ,s} \right)\, + \, \frac{{\sigma_{\text{s}} }}{4\pi }\int {I\left( {\hat{s},\hat{s}^{\prime}} \right)\varPhi \left( {g,\theta } \right){\text{d}}\varOmega },$$

where *I* denotes the light intensity at any point inside the reactor and at any algal concentration. *k* and σ_s_ are the effective absorption and scattering coefficients, respectively, which include the absorption and scattering by microalgae as well as scattering by air bubbles present in the PBR. Both absorption and scattering coefficients depend on the wavelength *λ* of the incident light and on concentration *c* in the following way:

5b $$\kappa \, = \,\bar{A}_{{{\text{abs}},\lambda }} c$$

5c $$\sigma_{\text{s}} \, = \, \bar{S}_{{{\text{sca}},\lambda }} c,$$

where $\bar{A}_{{{\text{abs}},\lambda }}$ and $\bar{S}_{{{\text{sca}},\lambda }}$ are the absorption and scattering cross sections, respectively. In Eq. 5a, *s* is the distance from the irradiated side of the reactor; $\hat{s}$ and $\hat{s}^{\prime}$ are the unit vectors signifying the incoming and the outgoing light at any point in the PBR. θ is the angle between the incoming and outgoing light vectors at a particular point. The integration over the solid angle ${\text{d}}\varOmega$ provides a sum over a unit sphere for all the incoming and outgoing light intensities at a particular point. *Φ*(*g*, *θ*) is the scattering phase function and describes the angular distribution of scattered light. There are several methods to approximate the scattering phase function [31]. Here, the Henyey–Greenstein phase function [33] has been used:

5d $$\varPhi \left( {g,\theta } \right)\, = \, \frac{{1\, - \,g^{2} }}{{\left( {1\, + \,g^{2} \, - \,2g{ \cos }\theta } \right)^{1.5} }}$$

where *g* is the asymmetry parameter which indicates back, forward or isotropic scattering.

$$- \,1\, < \,g\, < \,0 :{\text{Back scattering}}$$

$$g\, = \,0 :{\text{Isotropic scattering}}$$

$$0\, < \,g\, < \,1 :{\text{Forward scattering}}$$

Microalgae are strongly forward scattering organisms with asymmetry parameters *g* close to 1. The value of *g* used in this study for *Chlamydomonas reinhardtii* was 0.98 [34].

## Results and discussion

The hydrodynamic and particle tracing simulations are independent from the light intensity simulations, as is evident from Eqs. 1–5. Hence, the corresponding simulations were performed separately. The hydrodynamic and particle tracing simulations were performed using COMSOL Multiphysics, which discretizes the Navier–Stokes equations using the finite element method (FEM). The light distribution was computed by solving the RTE with self-implemented MATLAB code. Both results were then combined to obtain information about the dynamic light intensity experienced by individual algal cells moving through the PBR. Finally, the dynamic light exposure was analyzed in terms of light/dark cycles.

### Hydrodynamics

**Fig. 1:**
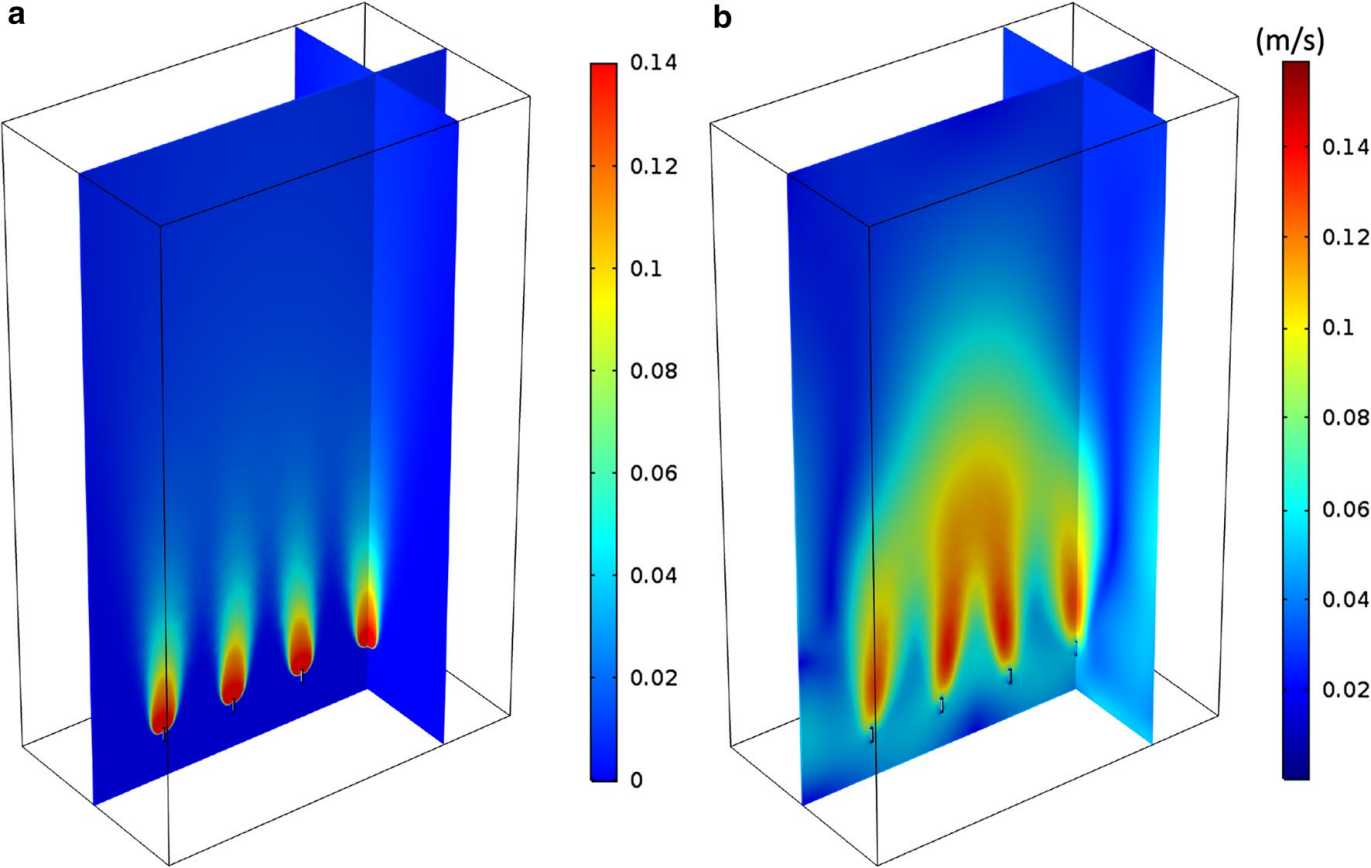
Results of hydrodynamic simulation in steady state, shown at two perpendicular planes in the PBR. **a** Gas phase volume fraction. **b** Liquid velocity magnitude

In the hydrodynamic simulations, the liquid is initially at rest and starts moving in loops due to the drag force applied by the air bubbles. Results of the calculation are the velocity profiles and the volume fractions of liquid and gas phases in the PBR as well as the turbulent variables *k* and *ɛ*. This study is focused on the steady-state behavior in the PBR. However, convergence was sped up by performing time-dependent simulations for a short time span and then using the results as initial guesses for the stationary simulations. Figure 1 shows the steady-state gas phase volume fraction distribution and liquid velocity field over two perpendicular planes in the PBR for a characteristic gas flow rate of 0.5 L/min (8.33 × 10^−6^ m^3^/s). Table 1 lists values of maximum and volume average velocities of the liquid phase, average velocities of the gas phase, and average gas volume fraction for different gas flow rates. The low gas volume fractions justify our usage of a slip model for the gas phase velocity profiles and the assumption of negligible coagulation and breakage of bubbles. Figure 1a illustrates that the gas volume fraction is highest in the central plane above the sparger as expected. Figure 1b shows that the maximum liquid velocity also occurs in the central plane, where the drag force of the air bubbles is maximal, while it decreases towards the sides of the PBR. Due to the no-slip boundary condition, the liquid velocity is always zero at the walls. The average liquid velocities in the whole PBR are low compared to the maximum velocity since the region of high velocities is comparably small. The liquid velocity decreases towards the top of the PBR. The average gas velocity in the PBR is much higher than the average liquid velocity, thus creating a large drag force on the liquid. While average gas volume fraction increases linearly with gas flow rate, the average gas velocity is almost the same for all flow rates. The gas has maximum velocity at the inlet, increasing with higher flow rate.

**Table 1:** Results of hydrodynamic simulations in stationary state for different gas flow rates

Gas flow rate		Liquid phase		Gas phase	
(L/min)	(10^−6^ m^3^/s)	Max. velocity (m/s)	Avg. velocity (m/s)	Avg. velocity (m/s)	Avg. volume fraction
0.01	0.167	0.0400	0.0027	0.2547	0.00010
0.25	4.167	0.1443	0.0211	0.2545	0.00226
0.5	8.333	0.1592	0.0288	0.2544	0.00430
0.75	12.50	0.1752	0.0329	0.2542	0.00642
1.0	16.67	0.1813	0.0365	0.2541	0.00857

From the hydrodynamic results, information can be extracted about the existence and size of dead zones in the PBR, where algal cells might be trapped and exposed to disadvantageous conditions. In this study, dead zones were defined as regions with liquid velocity less than 5% of the volume average velocity magnitude, which is approximately 1% of the maximum liquid velocity. With this definition, dead zones were observed only in very small regions (less than 2 mm^3^ volume) compared to the overall PBR volume, at each corner of the PBR. A possible cause for this insignificance of dead zones is the position of the gas inlet pipe, high enough in the PBR to allow the liquid to move in loops at the bottom of the reactor.

### Particle tracing

Trajectories of algal cells were calculated over time based on the previously computed liquid velocity field. As these velocities are averaged properties of a turbulent system, a turbulent dispersion term was added to the liquid velocity profile, i.e., a fluctuation was added to the average liquid velocity to account for local turbulence acting on the particles. This fluctuation was determined using the turbulent kinetic energy calculated during the hydrodynamic simulation. For statistical analysis, the particle tracing was performed for 10,000 algal cells and a time period of 100 s, with initial positions randomly distributed over the entire PBR. The number of particles and the simulation time were chosen such that the results do not change qualitatively if more cells and/or longer traces are used.

**Fig. 2:**
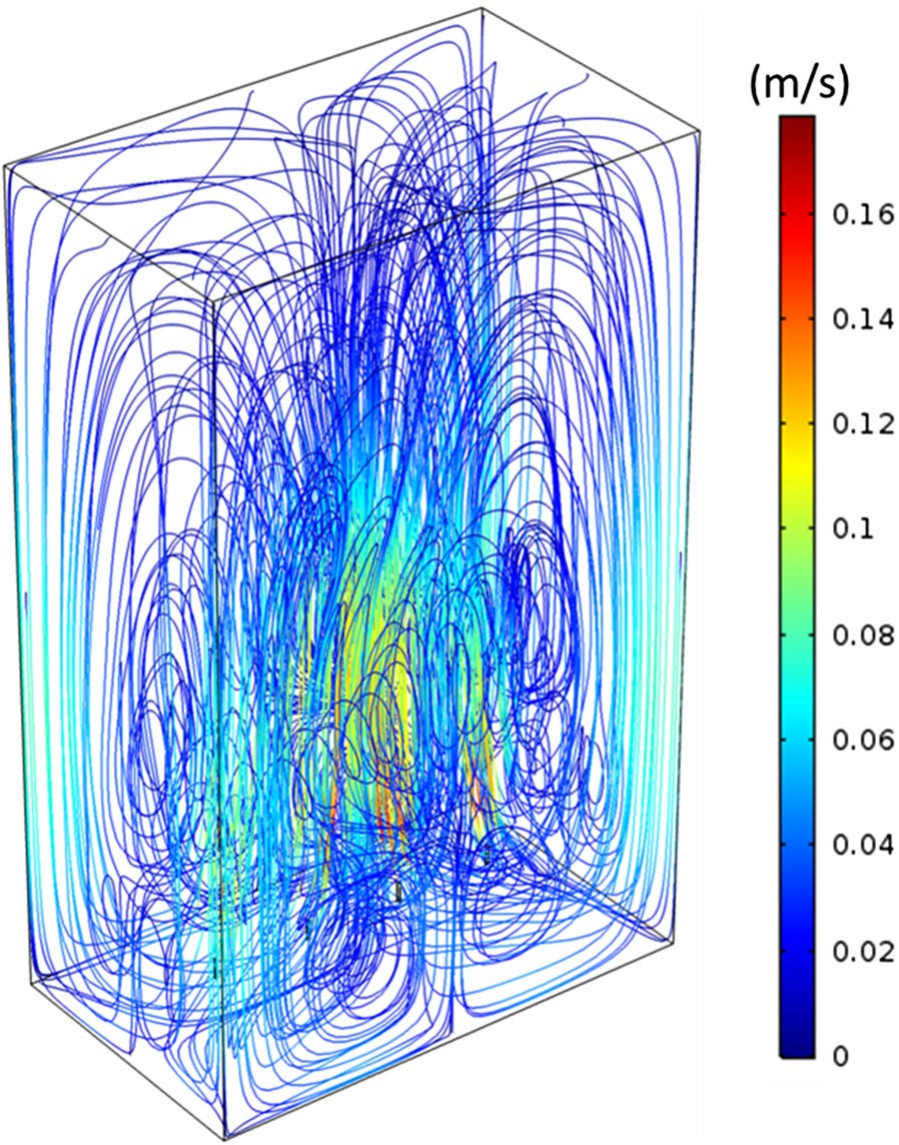
Traces of 27 algal cells for 100 s, starting from a uniform distribution in the PBR. Color indicates cell velocity

Figure 2 shows 27 example algal cells that were traced for 100 s. For better visualization, these traces were calculated without turbulent dispersion and from a uniform distribution of starting positions. The color of a trace illustrates the velocity of the respective cell as it moves through the PBR. The liquid velocity field used was the same as shown in Fig. 1, with a gas flow rate of 0.5 L/min (8.33 × 10^−6^ m^3^/s). The results shown in Fig. 2 indicate that the algae migrate through the entire reactor, and confirm that dead zones are practically not present in the studied PBR as no algal cells are trapped in any region for long times.

Most studies of algae movement in PBRs assume the algal cells to be distributed homogeneously in the PBR [7, 13, 31, 38]. Pruvost et al. [19] demonstrated that the Lagrangian approach used to define the movement of the algal cells leads to a numerical artefact: the number of cells along the walls of the PBR seems to be increased compared to the interior of the reactor. This is also the case in the present study. For an energetically consistent analysis of light absorption in the reactor, the seeming concentration inhomogeneity can be compensated by introducing locally heterogeneous light absorption rates [19]. Here, absorption rates have been kept constant in the calculation of the light distribution inside the reactor, and the effect of the artefact is discussed later on in the context of the dynamic light exposure of single cells.

### Light intensity

**Fig. 3:**
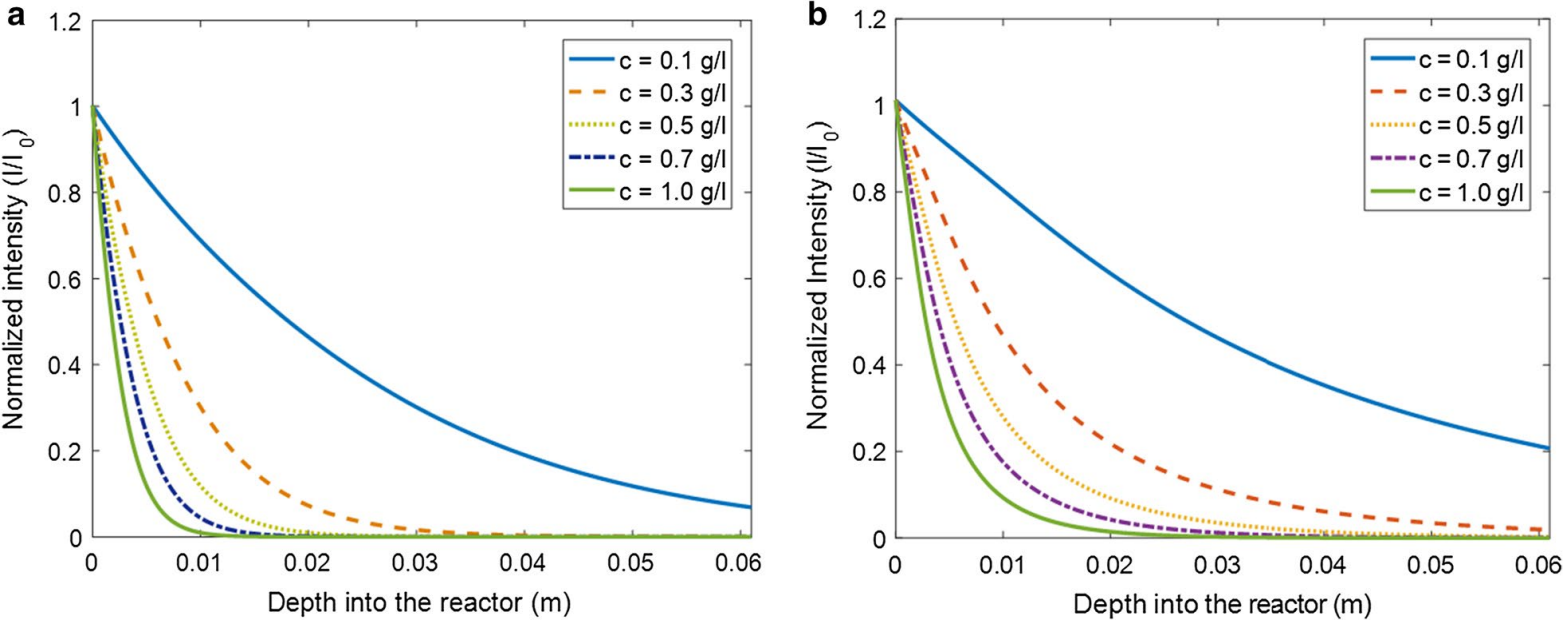
Variation of light intensity over the distance from the front PBR wall at different concentrations of algae obtained by solving the radiative transfer equation (RTE). **a** Blue and red incident light (485 and 670 nm); **b** White incident light (solar spectrum). Absorption and scattering coefficients were taken from [28] for algal strain *C. reinhardtii*

Incident light (*I*_0_) is absorbed and scattered by the algal cells and the air bubbles, creating a non-uniform distribution of light intensity inside the PBR. The most simple and wide-spread method to simulate the decay of light into the system is using Lambert–Beer’s law (LBL) [23]. It describes an exponential decay of light due to absorption by algae with increasing cell concentration and increasing distance from the irradiated PBR surface. Even though LBL does not account for the scattering of light by air bubbles or algal cells, it was found to be sufficient for practical purposes in many cases due to the mainly forward scattering properties of microalgae [35]. However, attenuation of green light is strongly affected by scattering because of the very high ratio of scattering and absorption cross sections in this case. Thus, and since the present study wants to establish a framework for computational analysis of light spectra with all wavelengths, in this study light intensities inside the PBR were calculated by numerically solving the RTE, Eq. 5a, which is an integro-differential equation, in MATLAB. The integral part, i.e., the in-scattering term, was solved using the Lebedev Quadrature technique [36, 37]. Figure 3 compares solutions of the RTE at different algae concentrations and wavelengths of irradiated light. Here, the depth into the PBR is the only spatial parameter along which the light intensity was calculated, since the incident light was assumed to be uniformly distributed across the PBR surface, boundary effects at the side walls of the PBR were neglected, and algal concentration differences along the width and height of the PBR were averaged for simplicity. Figure 3a was calculated with the same combination of blue and red light as often used in experiments performed on the PBR simulated here, i.e., 50% red and 50% blue [20, 21]. Figure 3b shows the distribution of light when irradiated with a solar spectrum in the PAR region (400–700 nm). Scattering and absorption by air bubbles were taken into account, but was observed to be negligible compared to contributions by algae. The scattering and absorption cross sections reported by [34] for algal strain *Chlamydomonas reinhardtii* were used to estimate the absorption and scattering coefficients in this study. These cross sections depend on wavelength of light and algal concentration. For blue light (wavelength 485 nm), the scattering and absorption cross sections are 872 and 386 m^2^/kg, while for green light (wavelength 535 nm) these cross sections are 1656 and 88 m^2^/kg, respectively [34]. Higher cell concentrations increase the effective absorption and scattering coefficients (see Eqs. 5b and 5c), thus increasing the attenuation of light. Figure 3a, b shows a strong difference of light attenuation of white light compared to blue and red light. This difference is mostly due to the green part (wavelengths between 530 and 570 nm) of the light spectrum, where values of the absorption coefficients are much lower than for blue and red light. Consequently, the proportion of the green part of the light in Fig. 3b changes from 14% at the reactor surface to 67% at the back of the reactor. Since the further focus of this study was on the simulation of a laboratory PBR illuminated by LEDs, only results for blue and red light (Fig. 3a) were used in the following.

As illustrated in Fig. 3a, some light reaches the other end of the PBR at an algal concentration (dry mass) of less than 0.16 g/L because the absorption and scattering coefficients are low. Concentrations of 0.3 g/L and above lead to a total consumption of the incident light within 4 cm or less in depth of the PBR, while algal cells in the remaining part of the PBR are in total darkness. Cornet [17] demonstrated that PBR performance is optimal if the working illuminated fraction, defined as “the part of the reactor volume having local irradiances higher than the compensation point for photosynthesis” is equal to 1. Since this theoretical condition is difficult to achieve and to maintain, especially in applications with fluctuating light, the present investigation deals also with scenarios where the working illuminated fraction is below 1. For further analysis, the reactor can be divided into two regions, light and dark zones, based on light availability. The light zone was defined as the region with normalized intensity (*I*/*I*_0_) greater than 0.02, because the specific growth rate of *Chlamydomonas reinhardtii* is positive for normalized light intensity greater than 0.02 [19], for an incident light intensity of 1000 µE/m^2^s. The rest of the reactor was defined as dark zone. At low concentrations (< 0.1 g/L), the light zone extends almost throughout the entire PBR, and it reduces to 20% (and less) at high concentrations (> 0.7 g/L).

**Fig. 4:**
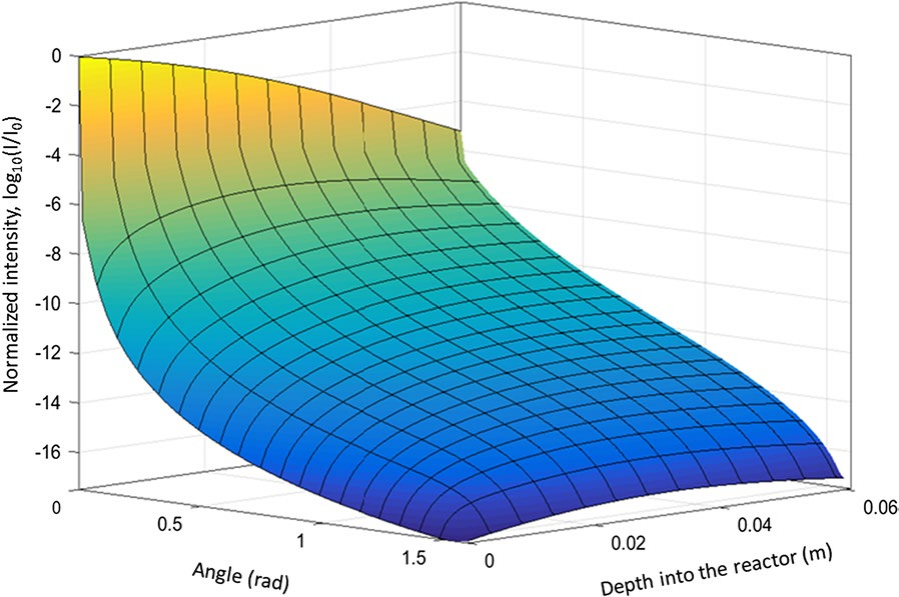
Angular distribution of light intensity in the reactor at algal cell concentration 0.1 g/L for light of wavelength 485 nm irradiated perpendicularly to the PBR surface

Light can be scattered in any direction by the algal cells. To account for the scattering in different angles, the Henyey–Greenstein phase function, Eq. 5b, was used which provides a good compromise between accuracy and computational complexity [31]. Figure 4 shows the angular distribution of light intensity, which is not considered by the LBL. At the surface of the PBR, all light is assumed to be irradiated in normal direction, but inside the PBR the light gets scattered in different angles and thus the intensity decreases in forward direction (0 radians in Fig. 4) while it increases in other directions. However, the algal cells scatter light mainly in forward direction, indicated by an asymmetry parameter *g* near 1 in Eq. 5b. In addition, the light intensity decreases along the depth of the reactor due to absorption by the microalgae. This explains the increase in light intensity in all directions directly behind the PBR surface and the flattening or decrease in deeper layers.

**Fig. 5:**
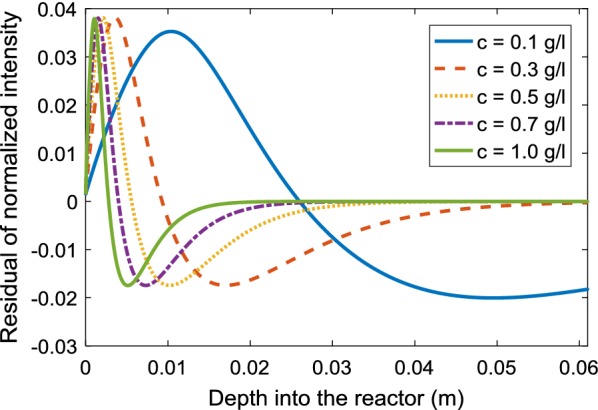
Residual of the light intensity from fitting a Lambert–Beer’s law (LBL) to the result of the radiative transfer equation (RTE) at different average algae concentrations for a combination of blue (485 nm) and red (670 nm) light

There have been approaches to amend the LBL by replacing the absorption coefficient with an effective coefficient in order to include the effect of light scattering [38, 39]. Here, this procedure was performed by adjusting the absorption coefficient of the standard LBL such that the light intensity profile approaches the profile I_RTE_ calculated using the RTE. The resulting optimized intensity I_LBL_, or I_LBL_^opt^, was then compared with I_RTE_. Figure 5 shows the difference of I_RTE_ and I_LBL_^opt^ at various algal concentrations. This difference is highest in the front region (i.e., at low depth into the reactor), where the absolute values of light intensities are high, too. This demonstrates that even the optimized LBL cannot correctly reproduce the effects of light scattering, making the application of the RTE necessary.

### Dynamic light exposure of single cells

**Fig. 6:**
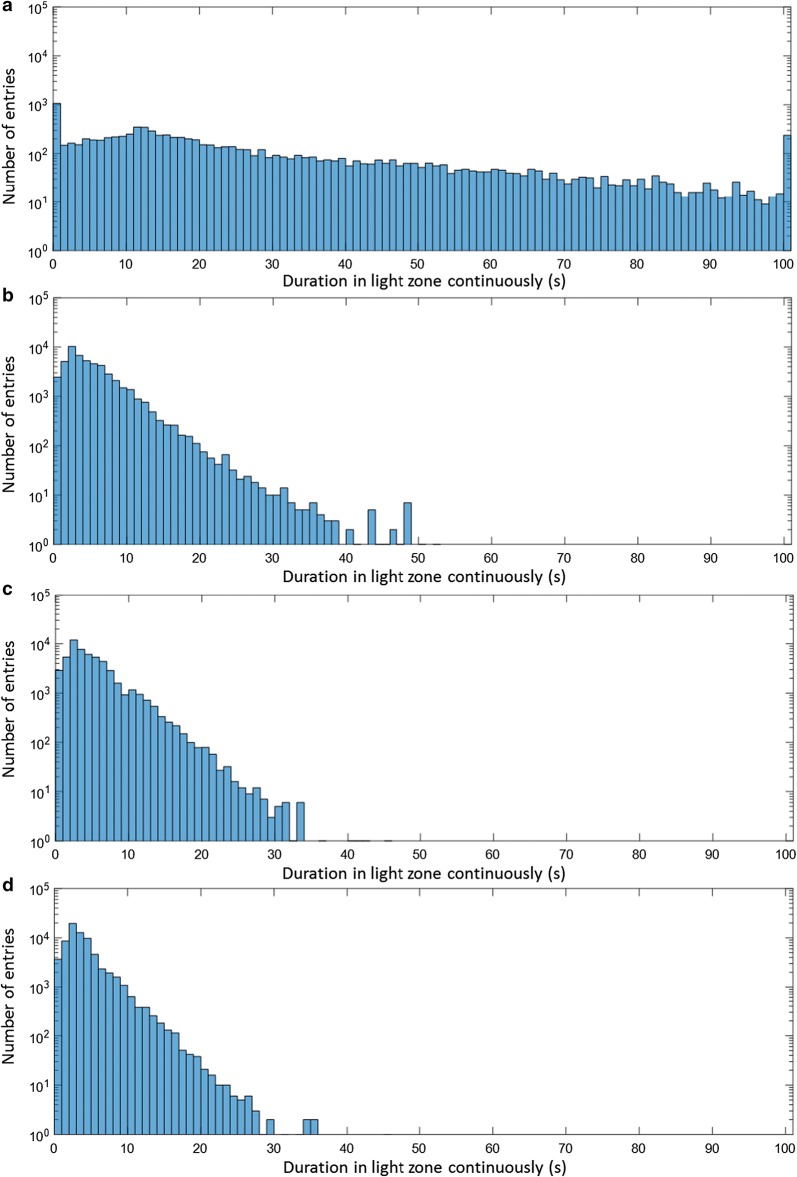
Number of visits to the light zone, sorted by their duration, computed from 10,000 particle traces each with a total simulation time of 100 s for combined red and blue light, an algal concentration of 0.5 g/L and a gas flow rate of **a** 0.01 L/min (0.167 × 10^−6^ m^3^/s), **b** 0.25 L/min (4.167 × 10^−6^ m^3^/s), **c** 0.5 L/min (8.33 × 10^−6^ m^3^/s) and **d** 0.75 L/min (12.5 × 10^−6^ m^3^/s)

**Fig. 7:**
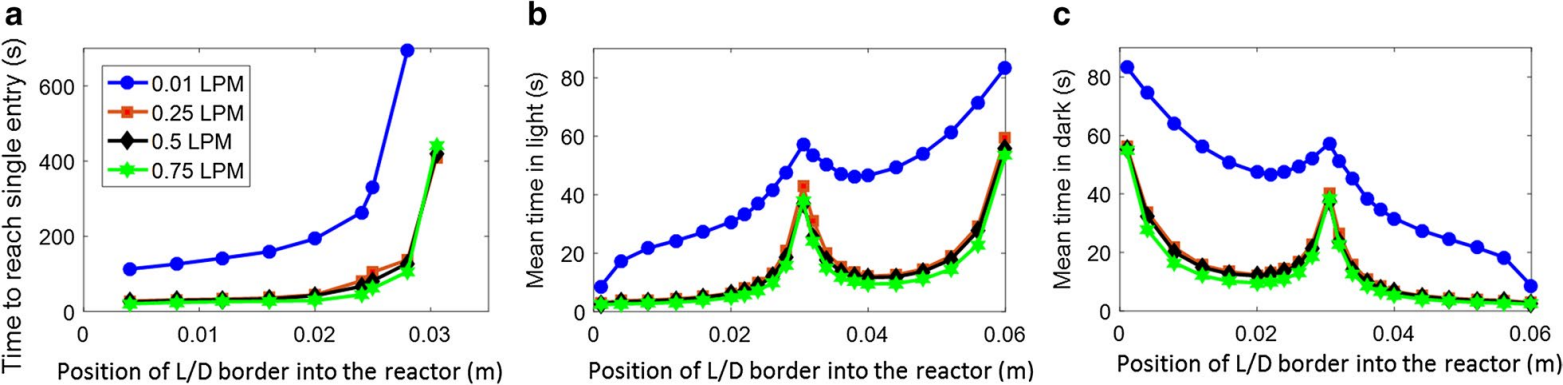
**a** Time at which the trend line fitted for histograms in Fig. 6 reaches a single entry, **b** mean time spent in light continuously and **c** mean time spent in dark continuously for different positions of light/dark border caused by different algal concentrations

Computation of light intensity and algal cell traces, i.e., the position of algal cells in the PBR over time, are independent of each other, and hence both were calculated independently and then combined to obtain the dynamic light exposure of individual cells over time. The impact of hydrodynamics on the light exposure is demonstrated in Fig. 6, which shows how long algal cells continuously stay in the light zone of the studied PBR before visiting the dark zone. These histograms were computed from the traces of 10,000 particles over 100 s at gas flow rates of 0.01, 0.25, 0.5, and 0.75 L/min (0.167, 4.167, 8.33, and 12.5 × 10^−6^ m^3^/s) for an algal concentration of 0.5 g/L. Figure 6 reveals that more than 50% of the visits to the light zone were shorter than 5 s for all higher flow rates, i.e., flowrates 0.25, 0.5, and 0.75 L/min, (note that the y-axis in Fig. 6 is shown on logarithmic scale). As the gas flow rate increases, the number of visits to the light zone increases, thus increasing the frequency of light/dark cycles, and the maximum time as well as the average time spent in the light decrease. The histograms in Fig. 6 clearly show the difference between the distributions when the gas flow rate is increased from 0.01 to 0.25 L/min, whereas the distributions change only slightly with further increase of the flow rate. The frequency of algal cells staying in the light zone continuously with the highest number of entries (between 2 and 3 s) remains practically the same for flow rates 0.25–0.75 L/min, signifying that the change from flow rates 0.25–0.75 L/min does not affect the dominant frequency of algal cells in the PBR. To further confirm this point, the following analysis was performed on the histograms shown in Fig. 6: the approximately exponential decrease (linear decrease in logarithmic scale, as shown in Fig. 6) of the number of entries over time for all the histograms was fitted with an exponential trend line (straight line on logarithmic scale) and the time where the number of entries decrease to one was approximated as a measure of maximal time spent in the light. This was performed for different concentrations of algae in the PBR, and results are shown in Fig. 7a. With increasing concentration the border between light and dark zones shifts from higher to lower depth into the PBR, thus the location of this border in the PBR is used in Fig. 7 instead of concentration, i.e., the lowest depth of the light/dark border refers to the highest concentration. It is shown in Fig. 7a that on shifting the light/dark border from lower to higher depth, the trend lines have a lower slope (in logarithmic scale) and thus the maximum time spent in the light increases. It is also clearly visible that there is almost no difference between the flow rates 0.25 and 0.75 L/min while these results are quite different from the flow rate 0.01 L/min. Figure 7b, c shows the mean times spent in the light and the dark zones continuously for all flow rates at different algal concentrations. For the flow rate of 0.01 L/min, the mean time spent in the light is higher than for the other flow rates at any concentration, which is desirable, but the mean time spent in the dark zone is high too, which leads to lower growth rates. Therefore, higher flow rates are desirable, because they reduce the average times spent in dark zones and increase the frequencies of light/dark cycles. In Fig. 7b, c, the symmetry of the reactor, even though blurred by the random turbulence of the algae cells, is still visible: algae move in loops in the front and back half of the reactor, respectively, with only limited exchange (see also Fig. 2). This causes the average time spent in the light to increase if the light/dark border is close to the middle of the reactor. All of the results of Fig. 7 are similar for the flow rates 0.25, 0.5, and 0.75 L/min while quite different for flow rate 0.01 L/min. The conclusion is that, for the present reactor design, a further increase of gas flow rates cannot improve mixing throughout the reactor. Other options would be more appropriate to prevent long presence of algal cells in the dark: illumination of the PBR from both sides would avoid the asymmetry of light distribution (Fig. 4) in the otherwise symmetrically arranged PBR; or changes to the reactor geometry, for example by moving the sparger inlet from the center of the PBR to one side, might create more favorable loops of fluid movement covering the full reactor volume. These are subject of future research.

## Conclusions

Algae cultivation was comprehensively studied by combining simulations of hydrodynamics, light distribution, and particle tracing in a flat-panel PBR. Single-cell residence times in light and dark zones are helpful to investigate under which algal concentrations and sparging rates the studied bioreactor is best operated in order to facilitate maximal utilization of the incident light. Hydrodynamic simulations allowed ruling out the existence of dead zones in the studied reactor design. Increasing the air sparging rate helps to avoid inactivation of the photosynthetic system by shortening the residence times of each visit in both dark and light zones. However, increasing the air sparging rate cannot overcome limitations of mixing caused by the reactor design. Based on these results, different approaches to improve mixing in the reactor were proposed. Future studies will apply metabolic growth models that take the history of light exposure into account, in order to study inactivation and inhibition of the photosynthetic system and their impacts on bioreactor design and operation in detail. The present contribution lays the foundation for such studies by providing single-cell traces with information on the environmental history.
